# Acute Pericarditis: An Isolated Initial Manifestation of Systemic Lupus Erythematosus (SLE)

**DOI:** 10.7759/cureus.97005

**Published:** 2025-11-16

**Authors:** Ulises Gomez-Alvarez, Carlos Gerardo Vargas Torres, Liliana Sanchez Soberanes, Juan Manuel Grajeda Marin, Jorge Luis Mora Castrejon

**Affiliations:** 1 Internal Medicine, Hospital General Instituto de Seguridad y Servicios Sociales de los Trabajadores del Estado (ISSSTE), Santiago de Querétaro, MEX

**Keywords:** acute pericarditis, autoimmune pericarditis, cardiac mri, systemic lupus erythematosus, young adult case

## Abstract

Pericarditis is an inflammation of the pericardium that may arise from multiple etiologies, including autoimmune diseases such as systemic lupus erythematosus (SLE). Although cardiovascular involvement is relatively common in established SLE, acute pericarditis as an isolated and initial manifestation is rare and can delay diagnosis. Constrictive pericarditis, although an uncommon feature of lupus, has been rarely reported as an initial presentation without prior recurrent episodes of acute pericarditis, further underscoring the heterogeneity of pericardial involvement in SLE. We present the case of a 23-year-old male with no significant past medical history who arrived at the ED with acute precordial chest pain radiating to the neck and back, exacerbated by leaning forward, and accompanied by tachycardia, dyspnea, and fever. On admission, the ECG demonstrated ST-segment elevation in leads V2-V5 and first-degree atrioventricular block, while inflammatory markers were elevated with normal troponin levels. A pericardial friction rub was noted on physical examination, and cardiac MRI showed evidence of acute pericardial inflammation. During hospitalization, acute kidney injury of suspected primary renal origin prompted autoimmune testing, which revealed high-titer antinuclear antibodies, positive anti-dsDNA and anti-SSA antibodies, and hypocomplementemia, confirming the diagnosis of SLE. The patient was initially managed with nonsteroidal anti-inflammatory drugs and colchicine and subsequently treated with prednisone, mycophenolate mofetil, and hydroxychloroquine, achieving clinical improvement and stabilization without recurrence of symptoms. This case illustrates the importance of considering autoimmune etiologies in young patients presenting with idiopathic pericarditis. Early identification of SLE in such atypical presentations allows timely initiation of immunosuppressive therapy, reduces the risk of severe complications such as lupus nephritis or cardiac tamponade, and highlights the value of a multidisciplinary approach to improve prognosis.

## Introduction

Pericarditis is an inflammatory condition of the pericardium that may arise from a wide variety of etiologies, ranging from viral infections to autoimmune disorders. Among the latter, systemic lupus erythematosus (SLE) is a chronic autoimmune disease characterized by multisystem involvement, most frequently affecting the skin (up to 80% of cases), joints (70-80%), kidneys (40-60%), and hematologic system (40%). Cardiovascular manifestations occur in approximately 25% of patients during the disease course, a finding that is particularly relevant given the significant impact of cardiac involvement on morbidity and mortality in SLE [1]. While pericarditis is a relatively common finding in established SLE, its occurrence as an initial and isolated manifestation is uncommon, representing less than 3% of reported cases [2]. Only a limited number of case reports have described pericarditis as the first presentation of lupus, underscoring its rarity and the diagnostic challenge it poses. This atypical presentation may contribute to diagnostic delay and increase the risk of severe complications, including pericardial effusion, cardiac tamponade, and lupus nephritis [3,4]. The evaluation of young patients with idiopathic pericarditis should therefore include a thorough search for autoimmune causes to ensure timely diagnosis and appropriate management [5]. In this report, we describe a case of acute pericarditis as the initial manifestation of SLE in a young male, highlighting the importance of integrating clinical, imaging, and serological findings for accurate diagnosis and optimal treatment.

## Case presentation

A 23-year-old male presented to the ED with acute precordial chest pain radiating to the neck and back, worsened by leaning forward, and accompanied by tachycardia. He subsequently developed dyspnea on moderate exertion and a fever measured at 38°C. On admission, his vital signs were as follows: blood pressure: 118/72 mmHg, heart rate: 108 beats per minute, respiratory rate: 20 breaths per minute, temperature: 38°C, and oxygen saturation: 97% on room air.

He had a family history of type 2 diabetes and systemic arterial hypertension, but no relevant chronic medical conditions. His social history included occasional alcohol use, minimal tobacco consumption (approximately one pack per week), and occasional marijuana inhalation. He denied recent infections, travel, biomass exposure, or contact with sick animals.

**Figure 1:**
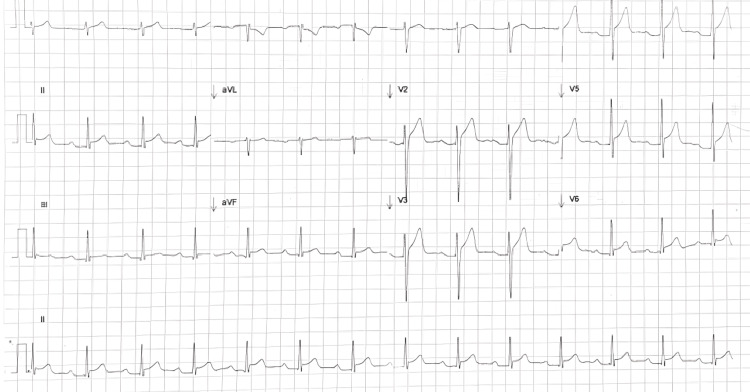
Admission ECG The ECG demonstrates concave ST-segment elevation in the precordial leads (V2-V5), which is characteristic of acute pericarditis. A prolonged PR interval (0.21 s) is also observed, consistent with a first-degree atrioventricular block.

On admission, the ECG showed ST-segment elevation in leads V2-V5 and first-degree atrioventricular block with a PR interval of 0.21 seconds (Figure 1).

On physical examination, a characteristic pericardial friction rub was auscultated. Laboratory evaluation demonstrated elevated inflammatory markers, including erythrocyte sedimentation rate and C-reactive protein, while cardiac biomarkers such as troponin remained within normal limits. Serum creatinine was also increased, consistent with acute kidney injury. A detailed summary of all laboratory and serologic findings is presented in Table 1.

**Table 1:** Laboratory results and normal ranges The laboratory findings, along with their reference ranges, are presented. They demonstrate elevated inflammatory markers and increased serum creatinine, consistent with acute kidney injury.

Laboratory test	Result	Normal range
Erythrocyte sedimentation rate	57 mm/h	0.0-15 mm/h
C-reactive protein	78 mg/dL	<1.0 mg/dL
Troponin I	14.96 pg/mL	<40.0 pg/mL
Serum creatinine	1.6 mg/dL	0.52-1.04 mg/dL

**Figure 2:**
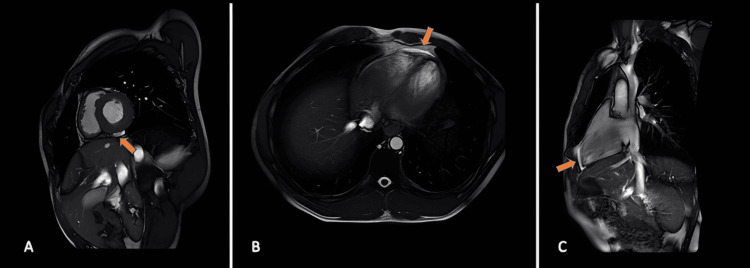
Cardiac MRI Different slices demonstrate hyperintensity of the left ventricular pericardium on T2-weighted sequences. (A) Short-axis view: The left ventricle is seen in cross-section, showing hyperintensity of the left ventricular pericardium on T2-weighted sequences (orange arrow). (B) Four-chamber view: The heart is visualized in a transverse plane, with both left and right ventricles visible. There is a 2 mm thickening of the pericardium with post-contrast enhancement on the T2 sequence (orange arrow). (C) Two-chamber view: The left ventricle and left atrium are seen along their full lengths, showing hyperintensity of the left ventricular pericardium (orange arrow).

During hospitalization, cardiac MRI demonstrated acute pericardial inflammation characterized by hyperintensity of the left ventricular pericardium in T2-weighted sequences, thickening of 2 mm, and post-contrast enhancement, consistent with early pericarditis. A transthoracic echocardiogram could not be performed due to technical limitations at the time; however, cardiac magnetic resonance provided high-resolution imaging that confirmed the diagnosis, as it offers greater sensitivity and specificity for detecting pericardial inflammation compared with echocardiography (Figure 2).

The presence of acute kidney injury that could not be explained by prerenal or postrenal causes raised suspicion for a primary renal process. Autoimmune testing was therefore undertaken, revealing high-titer antinuclear antibodies with a coarse speckled pattern. Subsequent serologic evaluation demonstrated positive anti-dsDNA and anti-SSA antibodies, along with hypocomplementemia, confirming the diagnosis of SLE. A detailed summary of the serologic findings is provided in Table 2.

**Table 2:** Serologic findings The patient exhibited high-titer ANA with a coarse speckled pattern, positive anti-dsDNA and anti-SSA antibodies, and low complement levels (C3 and C4), consistent with SLE. ANA, antinuclear antibodies; C3, complement component 3; C4, complement component 4; dsDNA, double-stranded DNA; SSA, Sjögren’s syndrome-related antigen A; SLE, systemic lupus erythematosus

Laboratory test	Result	Normal range
ANA	1:1280 Coarse speckled pattern	<1:80
Anti-dsDNA	558.99 IU/mL	<25 IU/mL (negative)
Anti-SSA (Ro)	200 U/mL	<20 U/mL (negative)
C3	56.11 mg/dL	90-180 mg/dL
C4	2.9 mg/dL	10-40 mg/dL

Initial treatment included nonsteroidal anti-inflammatory drugs and colchicine for pericarditis. After the diagnosis of SLE was established, oral prednisone was initiated at a dose of 1 mg/kg/day (60 mg daily), followed by a gradual taper of 10 mg every two weeks over a total of eight weeks. Mycophenolate mofetil (1 g twice daily) and hydroxychloroquine (200 mg twice daily) were also started.

**Figure 3:**
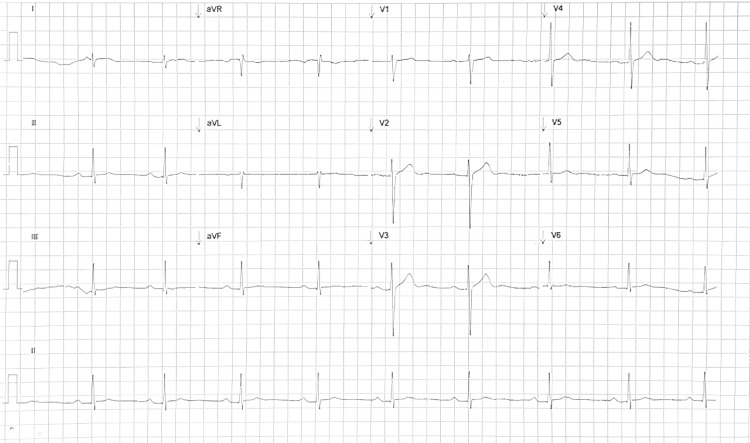
ECG at hospital discharge Resolution of ST-segment elevation in the precordial leads (V2-V5) and normalization of the PR interval.

During hospitalization, renal function progressively improved without requiring dialysis, and serum creatinine normalized before discharge, indicating resolution of the acute kidney injury. A renal biopsy was considered but deferred due to full recovery of renal function and absence of active urinary findings. The patient remained hospitalized for seven days and was discharged clinically stable and asymptomatic, with an ECG showing resolution of ST-segment elevation and normalization of the PR interval (Figure 3). He was referred to rheumatology for outpatient follow-up.

On follow-up, he remained asymptomatic and free of pericardial symptoms. Serial clinical and electrocardiographic assessments showed no signs of recurrence or constrictive pericarditis, and his overall cardiac function remained stable. The patient continued immunosuppressive treatment with mycophenolate mofetil (1 g twice daily), hydroxychloroquine (200 mg twice daily), and a tapering course of prednisone, which was discontinued after eight weeks. He remained asymptomatic and clinically stable on follow-up, with no recurrence of pericarditis or signs of lupus reactivation.

## Discussion

Pericarditis is a common cardiovascular manifestation in SLE, but its occurrence as an isolated initial presentation is rare, reported in less than 3% of cases [1]. This low frequency can contribute to diagnostic delays, underscoring the importance of a comprehensive evaluation in young patients with idiopathic pericarditis. In our case, early detection of antinuclear antibodies, followed by confirmation with anti-dsDNA and anti-SSA antibodies along with hypocomplementemia, was critical for establishing the diagnosis of SLE. These serologic markers have consistently been identified as key elements in cases of SLE-related pericarditis, supporting their inclusion in the diagnostic workup [2,3].

The use of complementary tools such as electrocardiography and cardiac MRI was essential to confirm pericardial inflammation and rule out alternative etiologies such as infectious or substance-induced conditions. Therapeutic management initially included nonsteroidal anti-inflammatory drugs and colchicine for symptomatic relief, later escalating to immunosuppressive therapy with corticosteroids, mycophenolate mofetil, and hydroxychloroquine once the diagnosis of SLE was confirmed. Early initiation of immunosuppressive treatment has been shown to be effective in preventing complications, including cardiac tamponade and lupus nephritis, and in improving long-term outcomes [4,5].

In addition to its role in evaluating pericardial inflammation, echocardiography remains a valuable tool for detecting other cardiac manifestations of SLE, even in asymptomatic patients. Previous studies have demonstrated a high prevalence of subclinical echocardiographic abnormalities, including valvular lesions (47.5%), pericardial effusion (13.6%), pulmonary artery hypertension (8.5%), pericardial thickening (6.8%), impaired systolic function (3.4%), and left ventricular hypokinesia (1.7%) [6]. Among these, Libman-Sacks endocarditis represents a distinctive nonbacterial valvular lesion characterized by sterile verrucous vegetations that rarely cause embolic events or hemodynamic compromise [1]. Although echocardiography was not performed in our patient, these findings underscore its importance in the comprehensive cardiac assessment of individuals with SLE.

Recent reports have documented that the spectrum of initial SLE manifestations can include isolated pericarditis, significant pericardial effusion, or even cardiac tamponade, highlighting the importance of considering SLE in the differential diagnosis of recurrent or persistent pericarditis [7,8]. Cardiovascular involvement remains a major cause of morbidity and mortality in SLE, and its timely recognition is crucial to improving prognosis [9,10]. Clinical experience also demonstrates that a multidisciplinary approach is essential for optimizing outcomes, with collaboration between rheumatology, cardiology, and internal medicine ensuring comprehensive care.

In Mexico, the true frequency of pericarditis as an initial manifestation of SLE remains unknown, emphasizing the need for further research and case documentation. This case contributes to the growing recognition of atypical presentations of SLE and reinforces the importance of thorough clinical, serological, and imaging assessment in patients presenting with acute pericarditis without an apparent cause.

## Conclusions

This case highlights pericarditis as an initial manifestation of SLE, emphasizing the importance of a comprehensive diagnostic approach in young patients with idiopathic pericarditis. The integration of clinical, serological, and imaging findings allowed early recognition of the underlying autoimmune disease and facilitated the prompt initiation of targeted therapy. Timely diagnosis and multidisciplinary management are essential to prevent severe complications and improve patient outcomes. Although constrictive pericarditis is an uncommon manifestation of lupus, it can occur as a sequela of recurrent acute pericarditis, underscoring the need for long-term cardiac monitoring in these patients. This report provides a practical example for the evaluation of similar cases and underscores the need for close follow-up in patients with atypical presentations of SLE.
